# Pre-Transplant C-Reactive Protein ≥ 20 mg/L Predicts Infection-Related Mortality After Heart Transplantation

**DOI:** 10.3390/jcm15041332

**Published:** 2026-02-08

**Authors:** Matthias Helmschrott, Karsten M. Heil, Rasmus Rivinius, Ann-Kathrin Rahm, Philipp Ehlermann, Norbert Frey, Fabrice F. Darche

**Affiliations:** Department of Cardiology, Angiology and Pneumology, Heidelberg University Hospital, 69120 Heidelberg, Germany

**Keywords:** cyclosporine A, immunosuppression, mortality, sepsis, tacrolimus

## Abstract

**Background**: Patients after heart transplantation (HTX) require lifelong immunosuppressive therapy to prevent graft rejection, thereby increasing susceptibility to infections. C-reactive protein (CRP) is a recognized biochemical marker of system-wide inflammation and generally rises with increasing infection severity. As the prognostic relevance of elevated CRP (≥20 mg/L) prior to HTX has been unclear, we analyzed its effects on post-transplant outcomes. **Methods**: We performed a retrospective, observational, single-center study including 418 patients who received HTX at Heidelberg Heart Center between the years 2000 and 2019. HTX recipients were grouped according to pre-transplant CRP (<20 or ≥20 mg/L). We analyzed donor and recipient characteristics, post-transplant pharmacotherapy, and post-transplant mortality including causes of death. **Results**: Pre-transplant CRP was ≥20 mg/L in 102 of 418 HTX recipients (24.4%). These patients had a significantly higher 30-day (11.8% versus 5.1%, *p* = 0.019), 1-year (39.2% versus 17.4%, *p* < 0.001), 2-year (42.2% versus 23.1%, *p* < 0.001), and 5-year post-transplant mortality (47.1% versus 30.4%, *p* = 0.002). Infection/sepsis was more frequently the cause of death within five years after HTX among patients with a pre-transplant CRP ≥ 20 mg/L (28.4% vs. 15.8%, *p* = 0.005), particularly pulmonary infections (19.6% vs. 9.5%, *p* = 0.006). Multivariate Cox regression showed pre-transplant CRP ≥ 20 mg/L as an independent predictor of 5-year post-transplant mortality (HR: 1.630, 95% CI: 1.144–2.323, *p* = 0.007). **Conclusions**: Pre-transplant CRP ≥ 20 mg/L identifies HTX candidates at increased risk of infection-related mortality after HTX, particularly pulmonary infections. Intensified pre-transplant evaluation for occult infection and close post-transplant infectious surveillance is advisable.

## 1. Introduction

Heart transplantation (HTX) is the ultimate therapeutic intervention for patients with refractory end-stage heart failure [1,2,3]. Despite major progress in surgical technique, pharmacotherapy, and multidisciplinary post-transplant care, HTX recipients continue to experience important complications, including acute rejection, graft failure, malignancy, and infectious events [4,5,6,7,8,9]. A central aspect of post-transplant management in HTX recipients is therefore the administration of immunosuppressive drug therapy to prevent graft rejection but this essential treatment substantially increases the risk of infectious complications [4,5,8,9].

Infectious complications remain a leading contributor to morbidity and mortality after HTX, especially in the early post-transplant period when immunosuppressive therapy is most intense [8,9]. Pneumonia and other pulmonary infections are particularly common and associated with high mortality in this context, reflecting both profound immunosuppression and the high vulnerability of respiratory tissues to opportunistic pathogens [8,9]. Clinical practice therefore emphasizes a low threshold for diagnostic testing (e.g., early imaging, microbiological sampling including blood cultures and respiratory specimens, and invasive diagnostics when needed) and prompt empiric antimicrobial therapy with subsequent pathogen-directed tailoring [10]. Beyond pneumonia, infective endocarditis is an uncommon but potentially fatal post-transplant infection [11]. Reported cases highlight substantial mortality, frequent diagnostic reliance on repeated blood cultures and echocardiography (often transesophageal), and a management strategy centered on prolonged intravenous antimicrobial therapy with surgical intervention in selected patients [11]. Because valve involvement can manifest as new or worsening valvular regurgitation, multimodality imaging approaches may be helpful when clinical suspicion persists despite initial testing [12].

In this setting, pre-transplant inflammatory activation may plausibly act as a vulnerability marker for severe infection after HTX. C-reactive protein (CRP) is a recognized biochemical marker of system-wide inflammation and commonly used in clinical practice. In patients with established cardiovascular disease, higher CRP levels have been linked to heart failure and adverse outcomes, consistent with the detrimental effects of systemic inflammation [13].

These findings are consistent with the broader transplant literature, in which pre-transplant CRP has been linked to adverse outcomes, including increased post-transplant mortality, in kidney, liver, and hematopoietic stem cell transplant populations [14,15,16]. In the context of HTX, elevated pre-transplant CRP has similarly been implicated as a potential marker of adverse post-transplant outcomes, such as higher mortality after HTX, although the available evidence remains limited [17].

In light of this limited evidence, the present study aimed to investigate the possible association between pre-transplant CRP ≥ 20 mg/L and post-transplant mortality, with a specific focus on infection-related mortality, particularly pulmonary infections. In short, we decided to use a pre-transplant CRP threshold of ≥20 mg/L because it reflects clinically meaningful systemic inflammation and no HTX-specific pre-transplant CRP threshold has yet been established [18,19,20]. Further details on why we chose this cut-off are provided in the methods.

## 2. Patients and Methods

### 2.1. Patients

This study adhered to the ethical principles of the Declaration of Helsinki. Ethics approval was issued by the institutional review board of Heidelberg University, Heidelberg, Germany (approval number S-286/2015, version 1.2, 28 July 2020). For the scientific use of routine clinical data, patients provided written informed consent for inclusion in the Heidelberg HTX Registry. As this retrospective, observational single-center analysis used routinely collected clinical information within the scope of the approved ethics protocol, no additional informed consent was required [21,22,23].

We reviewed and analyzed the medical records of the Heidelberg HTX Registry. For this study, we applied the following inclusion and exclusion criteria: Inclusion criteria: (I) Primary HTX at the Heidelberg Heart Center between 2000–2019; (II) HTX recipient age at the time of HTX ≥ 18 years; (III) Available pre-transplant CRP measurement obtained prior to HTX; (IV) Available 5-year post-transplant follow-up for survival status and cause of death. Exclusion criteria: (I) Repeat HTX; (II) HTX recipient age at the time of HTX < 18 years; (III) Missing pre-transplant CRP data; (IV) Missing 5-year post-transplant follow-up for survival status and cause of death.

For the present study, patients were categorized into two cohorts: patients with a pre-transplant CRP < 20 mg/L and patients with a pre-transplant CRP ≥ 20 mg/L. Pre-transplant CRP was routinely measured immediately prior to HTX. We defined a pre-transplant CRP ≥ 20 mg/L as clinically relevant for several reasons. First, to date, no HTX-specific pre-transplant CRP threshold has been established [18,19,20]. Second, to our knowledge, the only available reference for a pre-transplant CRP cut-off is a study of 43 HTX recipients, in which an optimal pre-transplant CRP threshold of 16.6 mg/L for predicting 6-month post-transplant mortality was identified using receiver operating characteristic (ROC) curve analysis and Youden’s index [17]. Third, to avoid overfitting and sample-specific bias inherent to data-driven cut-off determination, we deliberately refrained from performing an ROC analysis within our own cohort and instead applied a predefined threshold to enhance methodological rigor and external validity. Fourth, we chose a CRP threshold of 20 mg/L because it is a clinically established cut-off, including in the National Institute for Health and Care Excellence (NICE) guidance for pneumonia management in the general population, where CRP values < 20 mg/L are generally considered clinically insignificant and typically do not justify antibiotic therapy [24].

### 2.2. Follow-Up

Follow-up was conducted in accordance with the standard protocol of the Heidelberg Heart Center [21,22,23]. Following hospital discharge, HTX recipients attended the Heidelberg HTX outpatient clinic monthly for the first six months post-transplant, then every two months until the completion of the first post-transplant year. Thereafter, three to four appointments at the Heidelberg HTX outpatient clinic per year were typically scheduled, with additional visits arranged when clinically indicated [21,22,23]. Complete follow-up information was obtained for all HTX recipients [21,22,23].

Heidelberg Heart Center standard follow-up included medical history review, physical examination, electrocardiography (ECG), echocardiography, laboratory testing, immunosuppressive drug monitoring, and endomyocardial biopsy. Additionally, patients underwent an annual chest radiograph and 24-h Holter monitoring [21,22,23].

### 2.3. Post-Transplant Pharmacotherapy

Immunosuppressive drug therapy and concomitant medications after HTX were administered in accordance with the Heidelberg Heart Center standard protocol [21,22,23]. Induction therapy was routinely performed using anti-thymocyte globulin–based immunosuppression. Early in the study period, the initial maintenance regimen included cyclosporine A in combination with azathioprine. Regarding the changes in the immunosuppressive drug regimen during the study period, mycophenolic acid successively superseded azathioprine from 2001 onward, and tacrolimus progressively succeeded cyclosporine A from 2006 onward. According to the Heidelberg Heart Center standard protocol, corticosteroids (prednisolone) were routinely administered after HTX, gradually tapered over time, and finally stopped after the first half of the first post-transplant year, if medically viable [21,22,23].

### 2.4. Statistical Analysis

Statistical analyses were performed using MedCalc (Version 23.2.1, MedCalc Software Ltd., Ostend, Belgium). Continuous variables are presented as mean ± standard deviation (SD), and categorical variables as counts (*n*) with corresponding percentages (%). Measures of association are presented as mean differences with 95% confidence intervals (CI). Statistical testing was selected based on data distribution and study question and included analysis of variance (ANOVA), chi-squared test, Fisher’s exact test, Kruskal–Wallis test, Mann–Whitney U-test, or Student’s *t*-test, as appropriate. Inter-group survival after HTX was evaluated using Kaplan–Meier curves with logrank testing. We generated all figures with CorelDRAW Graphics Suite 2025 (version 26.2.0.170; Corel Corporation, Ottawa, ON, Canada). A *p*-value < 0.050 was deemed the threshold for statistical significance [21,22,23].

Comprehensive univariate analyses were performed to detect differences between patients with a pre-transplant CRP < 20 mg/L and patients with a pre-transplant CRP ≥ 20 mg/L. Examined variables covered recipient characteristics, history of open-heart surgery before HTX, primary indication for HTX, donor characteristics, donor–recipient sex mismatch, perioperative parameters, immunosuppressive therapy, and concomitant post-transplant pharmacotherapy [21,22,23].

The prespecified primary endpoint was 5-year survival following HTX, comparing patients with a pre-transplant CRP < 20 mg/L to those with a pre-transplant CRP ≥ 20 mg/L. Additional subgroup analyses of survival in accordance with immunosuppressive drug therapy were performed comparing 5-year post-transplant survival between patients on cyclosporine A with a pre-transplant CRP < 20 or ≥20 mg/L, between patients on tacrolimus with a pre-transplant CRP < 20 or ≥20 mg/L, as well as between patients with a pre-transplant CRP ≥ 20 mg/L on cyclosporine A or tacrolimus. All deaths occurring within five years after HTX were categorized as acute rejection, graft failure, thromboembolic event/bleeding, malignancy, or infection/sepsis. Infection/sepsis-related deaths were further classified as pulmonary or abdominal infections [22,23].

To evaluate independent predictors of 5-year post-transplant mortality, we fitted a Cox regression (multivariate analysis) using variables with univariate statistical significance: recipient chronic kidney disease, recipient estimated glomerular filtration rate (eGFR), ischemic time, and recipient CRP ≥ 20 mg/L before HTX. Secondary outcomes included early occurrence (≤30 days after HTX) of atrial fibrillation (AF), rejection episodes, transient ischemic attack (TIA), and stroke, comparing HTX recipients with a pre-transplant CRP < 20 mg/L versus ≥20 mg/L. Finally, to assess latent era effects across the extended study period, we performed a sensitivity analysis restricted to patients who received mycophenolic acid and tacrolimus, the standard immunosuppressive drug regimen implemented from 2006 onward [21,22,23].

## 3. Results

### 3.1. Demographic and Clinical Characteristics

After application of the exclusion criteria, 418 HTX recipients were included in the study. Of these, 316 patients (75.6%) had a pre-transplant CRP < 20 mg/L, while 102 patients (24.4%) had a pre-transplant CRP ≥ 20 mg/L.

Overall, baseline characteristics were similar between groups (Table 1). Patients with a pre-transplant CRP ≥ 20 mg/L more often had chronic kidney disease (67.6% versus 50.9%, *p* = 0.003), had a lower eGFR (52.7 ± 23.3 mL/min/1.73 m^2^ versus 61.8 ± 23.2 mL/min/1.73 m^2^, *p* = 0.001), and had a longer ischemic time (262.6 ± 61.7 min versus 247.6 ± 57.9 min, *p* = 0.032), whereas no other recipient, donor, or procedural variables differed significantly.

### 3.2. Post-Transplant Medications

Post-transplant immunosuppressive treatment and concomitant medications were comparable (all *p* ≥ 0.050) between HTX recipients with a pre-transplant CRP < 20 mg/L and those with a pre-transplant CRP ≥ 20 mg/L (Table 2).

### 3.3. Post-Transplant Primary Outcome

For the primary endpoint, HTX recipients with a pre-transplant CRP ≥ 20 mg/L had a significantly higher 30-day (11.8% versus 5.1%, *p* = 0.019), 1-year (39.2% versus 17.4%, *p* < 0.001), 2-year (42.2% versus 23.1%, *p* < 0.001), and 5-year post-transplant mortality (47.1% versus 30.4%, *p* = 0.002). Detailed information is presented in Table 3.

**Figure 1:**
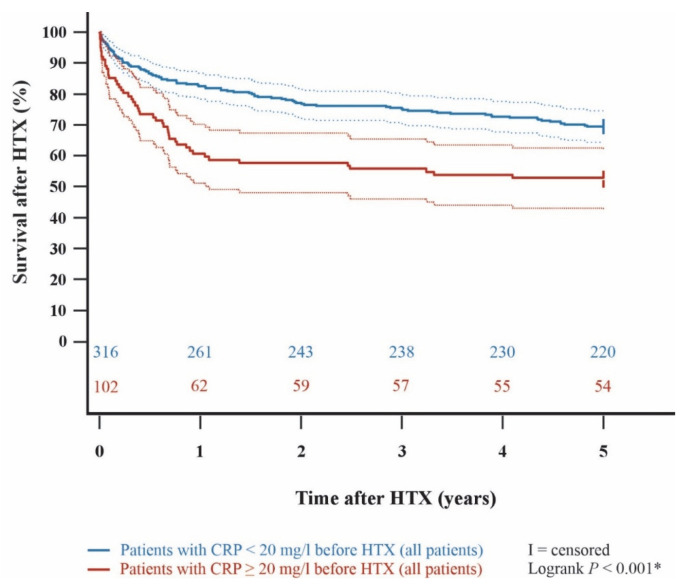
5-year post-transplant survival between patients with a pre-transplant CRP < 20 mg/L versus ≥20 mg/L (Kaplan–Meier curve). Patients with a pre-transplant CRP ≥ 20 mg/L showed a significantly decreased 5-year survival after HTX (*p* < 0.001). Dashed lines represent the 95% confidence interval around the respective survival curve. CRP = C-reactive protein; HTX = heart transplantation; * = statistically significant (*p* < 0.050).

**Figure 2:**
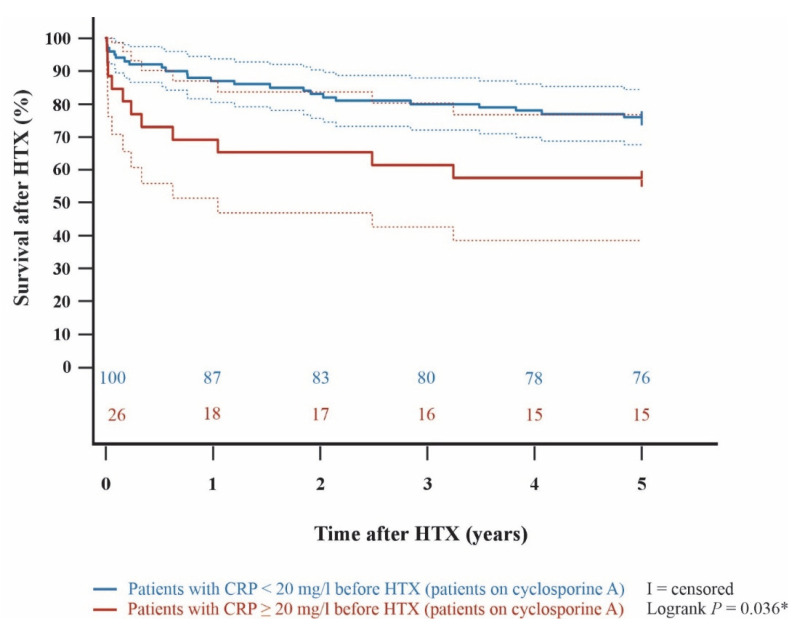
5-year post-transplant survival between patients on cyclosporine A with a pre-transplant CRP < 20 mg/L and patients on cyclosporine A with a pre-transplant CRP ≥ 20 mg/L (Kaplan–Meier curve). Patients on cyclosporine A with a pre-transplant CRP ≥ 20 mg/L showed a significantly decreased 5-year survival after HTX versus patients on cyclosporine A with a pre-transplant CRP < 20 mg/L (*p* = 0.036). Dashed lines represent the 95% confidence interval around the respective survival curve. CRP = C-reactive protein; HTX = heart transplantation; * = statistically significant (*p* < 0.050).

**Figure 3:**
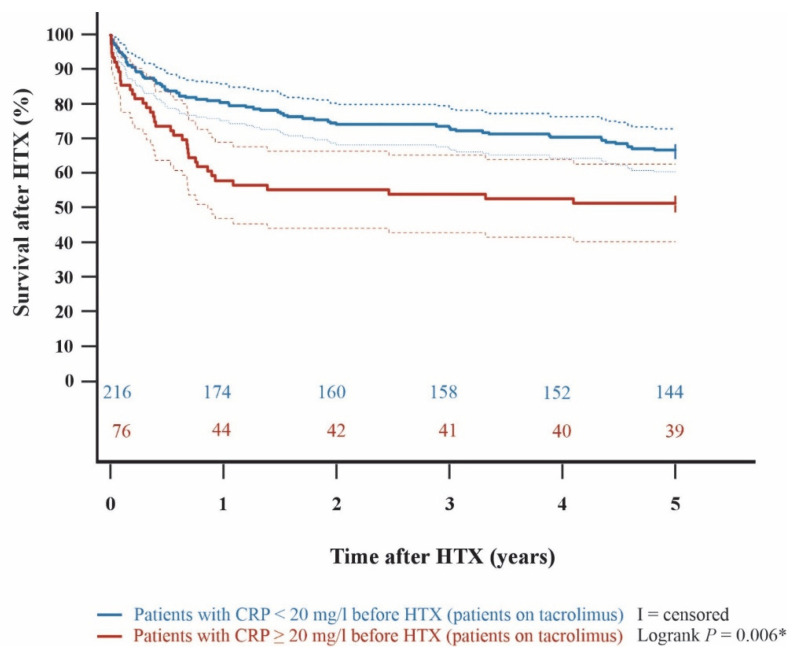
5-year post-transplant survival between patients on tacrolimus with a pre-transplant CRP < 20 mg/L and patients on tacrolimus with a pre-transplant CRP ≥ 20 mg/L (Kaplan–Meier curve). Patients on tacrolimus with a pre-transplant CRP ≥ 20 mg/L showed a significantly decreased 5-year survival after HTX versus patients on tacrolimus with a pre-transplant CRP < 20 mg/L (*p* = 0.006). Dashed lines represent the 95% confidence interval around the respective survival curve. CRP = C-reactive protein; HTX = heart transplantation; * = statistically significant (*p* < 0.050).

**Figure 4:**
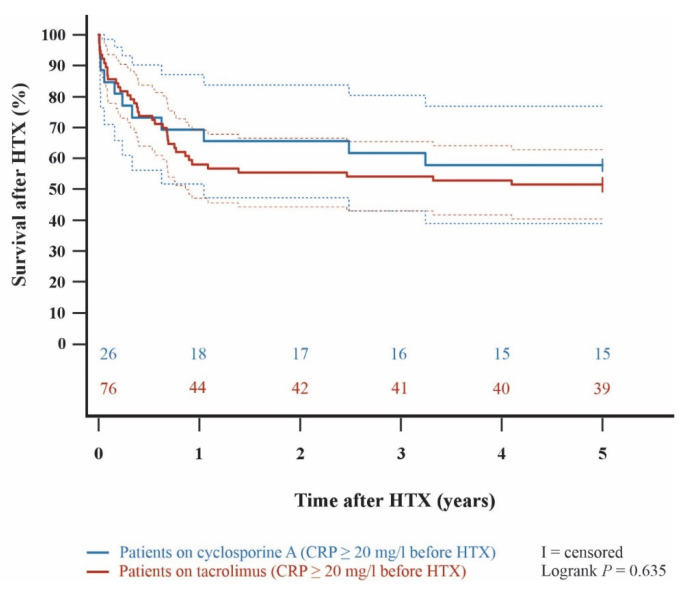
5-year post-transplant survival between patients with a pre-transplant CRP ≥ 20 mg/L on cyclosporine A and patients with a pre-transplant CRP ≥ 20 mg/L on tacrolimus (Kaplan–Meier curve). There was no significant between-group difference in 5-year survival after HTX (*p* = 0.635). Dashed lines represent the 95% confidence interval around the respective survival curve. CRP = C-reactive protein; HTX = heart transplantation.

Kaplan–Meier analysis demonstrated an impaired 5-year post-transplant survival (*p* < 0.001) in HTX recipients with a pre-transplant CRP ≥ 20 mg/L in comparison to HTX recipients with a pre-transplant CRP < 20 mg/L. In subgroup analyses, 5-year post-transplant survival was lower in cyclosporine A-treated HTX recipients with pre-transplant CRP ≥ 20 mg/L than in those with CRP < 20 mg/L (*p* = 0.036). Likewise, patients on tacrolimus with a pre-transplant CRP ≥ 20 mg/L showed a significantly lower 5-year post-transplant survival than patients on tacrolimus with a pre-transplant CRP < 20 mg/L (*p* = 0.006). Among HTX recipients with a pre-transplant CRP ≥ 20 mg/L, 5-year post-transplant survival did not differ significantly between those treated with cyclosporine A versus tacrolimus (*p* = 0.635). Kaplan–Meier curves are shown in Figure 1, Figure 2, Figure 3 and Figure 4.

Analysis of causes of death within five years after HTX demonstrated a significantly higher percentage of infection/sepsis-related mortality among patients with pre-transplant CRP ≥ 20 mg/L (28.4% versus 15.8%, *p* = 0.005). Subgroup analysis revealed that this difference was primarily attributable to pulmonary infections, which accounted for a significantly higher proportion of deaths in patients with a pre-transplant CRP ≥ 20 mg/L (19.6% versus 9.5%, *p* = 0.006), whereas no significant difference in 5-year mortality after HTX was observed for abdominal infections between the two groups (8.8% versus 6.3%, *p* = 0.389). Furthermore, no significant differences in 5-year mortality after HTX were observed between the two groups in terms of acute rejection, graft failure, malignancy, or thromboembolic event/bleeding (all *p* ≥ 0.050). Details are provided in Table 4.

In multivariate Cox regression, pre-transplant CRP ≥ 20 mg/L was an independent predictor of 5-year mortality after HTX (HR: 1.630, 95% CI: 1.144–2.323, *p* = 0.007), whereas the other three included variables (recipient chronic kidney disease, recipient eGFR, and ischemic time) were not significant predictors of 5-year mortality after HTX. Results of the multivariate analysis are shown in Table 5.

### 3.4. Post-Transplant Secondary Outcomes

With respect to secondary outcomes, we observed no significant differences between HTX recipients with a pre-transplant CRP < 20 mg/L and HTX recipients with a pre-transplant CRP ≥ 20 mg/L in terms of 30-day atrial fibrillation after HTX (13.6% versus 15.7%, *p* = 0.543), 30-day rejection episode after HTX (13.6% versus 9.8%, *p* = 0.315), 30-day TIA after HTX (0.0% versus 0.0%), or 30-day stroke after HTX (2.8% versus 5.9%, *p* = 0.152). Post-transplant secondary outcomes are shown in Table 6.

### 3.5. Sensitivity Analysis

To account for the long inclusion period, we explored potential era effects by performing a sensitivity analysis restricted to HTX recipients treated with mycophenolic acid and tacrolimus as maintenance immunosuppression (292 of 418 patients [69.9%]). The findings were consistent with the primary analysis, thereby strengthening confidence in our study results and minimizing the probability of potential bias related to temporal changes in practice.

## 4. Discussion

### 4.1. Pre-Transplant CRP and Mortality After Heart Transplantation

In our cohort of 418 HTX recipients, a pre-transplant CRP ≥ 20 mg/L was strongly associated with higher post-transplant mortality, and this association persisted after multivariate adjustment. Patients with CRP above this threshold had an approximately 1.6-fold higher hazard of 5-year mortality after HTX independent of other risk factors. This finding aligns with and extends prior limited evidence of a small sample-size study in HTX recipients [17]. Huma and colleagues [17] found that a pre-transplant CRP > 16.6 mg/L (threshold derived from receiver operating characteristic curve and Youden’s index) was associated with markedly higher 6-month mortality after HTX (*p* < 0.01) supporting previous findings about an association between a pro-inflammatory milieu and increased cardiovascular risk in non-transplant patients [25,26,27].

Similar observations in other organ transplant cohorts reinforce this notion [14,15,16]. In renal transplantation, a pre-transplant CRP > 10 mg/L was associated with increased post-transplant mortality and remained the only significant predictor (*p* < 0.05) of post-transplant mortality in multivariate analysis [14]. In liver transplantation, a pre-transplant CRP > 30 mg/L was independently associated with reduced survival (*p* = 0.009) in multivariate analysis [15]. Likewise, in the setting of allogeneic hematopoietic stem cell transplantation, a meta-analysis found that elevated pre-transplant CRP is a significant prognostic marker for worse post-transplant survival, underscoring that baseline inflammation portends poor immune recovery [16]. These parallels across disciplines highlight the impact of elevated pre-transplant CRP on post-transplant mortality irrespective of the organ being transplanted [14,15,16,17].

Notably, our cohort showed that infection (especially pulmonary infection) was a predominant cause of death in HTX recipients with a pre-transplant CRP ≥ 20 mg/L, suggesting an association between pre-transplant inflammatory burden and susceptibility to post-transplant infections. This is of high clinical importance as infections are among the leading causes of morbidity and mortality after HTX [8,9,28]. For example, over 80% of HTX recipients developed at least one infection in the first six post-transplant months, with pulmonary infections being the most common type [9]. Similarly, in a multicenter study, Moayedi and colleagues [8] reported that the majority of post-transplant infections occurred within the first year after HTX.

Our finding that a pre-transplant CRP ≥ 20 mg/L is associated with infection-related mortality aligns with these data and supports the notion that an elevated inflammatory state at the time of HTX may identify patients at higher risk of life-threatening infections once immunosuppressed.

### 4.2. Pre-Transplant CRP and Immunosuppressive Drug Therapy

The strong association between pre-transplant CRP ≥ 20 mg/L and infection-related mortality after HTX is a central finding of this study. We observed that infection/sepsis was a disproportionately common cause of death in HTX recipients with a pre-transplant CRP ≥ 20 mg/L (28.4% versus 15.8% of deaths, *p* = 0.005) with this excess largely attributable to pulmonary infections (19.6% versus 9.5% of deaths, *p* = 0.006). This aligns with the well-recognized susceptibility of HTX recipients to opportunistic infections, especially in the early post-transplant period when immunosuppression is intense [8,9,28,29,30]. Pneumonia and other pulmonary infections are a major cause of early mortality after HTX [8,9], and our data suggest that those who receive HTX in a pro-inflammatory state are even more prone to fatal pulmonary infections. In fact, systemic inflammation per se has been associated with increased infection-related mortality [31]. Thus, an elevated CRP prior to HTX may signify an immunologically compromised host. It seems plausible that patients with a pre-transplant CRP ≥ 20 mg/L are colonized by undiagnosed pathogens or suffer from subtle pre-transplant infections due to pulmonary congestion. Following HTX, these patients are exposed to an additional burden from intensive immunosuppressive therapy superimposed on already impaired baseline immunity, creating a high-risk milieu for severe infections. In the lungs, inflammation-related endothelial and epithelial dysfunction might reduce barrier integrity and ciliary clearance, facilitating fatal pulmonary infections [8,9].

Mechanistically and pathophysiologically, an elevated pre-transplant CRP likely captures a composite risk state rather than a single pathway. It may reflect occult infection (including device-associated or respiratory infection in advanced heart failure), systemic inflammation from tissue congestion/ischemia, or immune dysregulation that precedes transplantation [8,9,28,29,30]. Following HTX, intense early immunosuppression may convert this pre-existing vulnerability into clinically overt, rapidly progressive infection, consistent with the observed enrichment of infection- and pulmonary infection–related deaths. The association between systemic inflammation and subsequent fatal infection has also been observed outside transplantation, supporting biologic plausibility [31].

Calcineurin inhibitors, such as cyclosporine A or tacrolimus, are the predominant maintenance immunosuppressive agents used in HTX recipients during the early post-transplant period [32,33,34,35]. Given the potential impact of differences between cyclosporine A and tacrolimus on post-transplant outcomes, we analyzed both agents with respect to pre-transplant CRP ≥ 20 mg/L. Importantly, the adverse impact of elevated pre-transplant CRP was observed despite comparable distribution of cyclosporine A and tacrolimus between patients with a pre-transplant CRP < 20 mg/L and those with a pre-transplant CRP ≥ 20 mg/L in our study. There were no differences in the use of induction or maintenance immunosuppressive drugs between the two groups. Furthermore, subgroup analyses confirmed that the adverse survival impact of a pre-transplant CRP ≥ 20 mg/L was consistent across HTX recipients treated with either cyclosporine A or tacrolimus as the main immunosuppressive agent, with both calcineurin inhibitor cohorts showing significantly reduced 5-year survival following HTX in patients with a pre-transplant CRP ≥ 20 mg/L. Moreover, within the pre-transplant CRP < 20 mg/L cohort, 5-year post-transplant mortality was comparable regardless of whether cyclosporine A or tacrolimus was used, indicating no statistically significant survival difference between the main immunosuppressive agents. These findings imply that the effects of pre-existing inflammation prior to HTX on post-transplant mortality cannot be mitigated simply by choosing one calcineurin inhibitor over another. Similarly, a previous study reported no difference in immunosuppressive regimen between HTX recipients with or without infectious complications after HTX [9].

On the other hand, while the immunosuppressive therapy predisposes HTX recipients to infectious complications, it is also essential to prevent rejection and graft failure [32,33,34,35]. Systemic inflammation and elevated CRP have been suggested to be associated with rejection and graft failure [36,37,38]. Eisenberg and colleagues [36] reported that elevated CRP is associated with graft failure (*p* = 0.003) and frequency of grade 3 rejection (*p* = 0.02) in HTX recipients. Likewise, Labarrere and colleagues [37] found CRP as a significant predictor of graft failure at 5 years (*p* = 0.005) and at 10 years after HTX (*p* = 0.003). In contrast, Battes and colleagues [38] observed no association between CRP and graft rejection after HTX.

We also found no significant differences in acute rejection or graft failure as causes of death within five years after HTX between patients with a pre-transplant CRP < 20 mg/L and those with a pre-transplant CRP ≥ 20 mg/L, implying that the excess mortality was not attributable to acute rejection or graft failure. Considering these complex circumstances, our study, as far as we are aware, represents the first extensive evaluation of the association between elevated pre-transplant CRP, post-transplant infections, and immunosuppressive drug therapy in HTX recipients, an area which has not yet been sufficiently explored [17].

### 4.3. Pre-Transplant CRP and Management After Heart Transplantation

In view of our findings and the clinical applications of our results, one could speculate that patients with markedly elevated pre-transplant CRP as a marker of pre-existing inflammation prior to HTX may benefit from closer surveillance for infections and individualized immunosuppression in the early post-transplant period. These observations may have important clinical implications for the management of HTX candidates and recipients. First, pre-transplant CRP ≥ 20 mg/L could be incorporated into risk stratification models to identify a subset of patients at substantially higher risk of infection-related mortality. Second, HTX candidates with a pre-transplant CRP ≥ 20 mg/L could benefit from thorough evaluation for occult infections, including dental, pulmonary, urinary, gastrointestinal, and device-related sources, with treatment, where feasible, prior to HTX. Sezgin and colleagues [39] demonstrated that, in HTX recipients, elevated CRP levels are associated with periodontitis, potentially contributing to systemic inflammation through oral pathogen translocation. This highlights the importance of good oral hygiene and regular dental care after HTX [39]. Even in the absence of detectable infection, a pre-transplant CRP ≥ 20 mg/L may serve as a signal to intensify optimization efforts, including intensified surveillance for infections before, during, and after HTX, consideration of broader or prolonged antimicrobial prophylaxis, closer monitoring for opportunistic infections, individualized prophylaxis strategies, and a lower threshold for diagnostic imaging and cultures in the setting of fever or respiratory symptoms, given the clinical importance of post-transplant infections [8,9,28,29,30].

Importantly, our findings highlight pre-transplant CRP ≥ 20 mg/L as a relevant clinical risk indicator, reflecting an active systemic inflammatory state. Identification of patients with a pre-transplant CRP ≥ 20 mg/L may enable targeted optimization strategies such as comprehensive infection screening, infection eradication, and individualized immunosuppression, with the potential to improve post-transplant outcomes. Future studies with prospective designs and standardized infection adjudication could clarify whether excess pulmonary infection mortality is mediated by specific pathogens, timing (early versus late), or modifiable post-transplant care processes. Moreover, analyzing pre-transplant CRP as a continuous variable and integrating additional inflammatory biomarkers (e.g., procalcitonin, interleukin-6) as well as clinical factors (e.g., mechanical circulatory support, colonization status) may improve risk discrimination and stratification. Ultimately, however, it remains uncertain whether lowering pre-transplant CRP, either by addressing its underlying causes or with anti-inflammatory interventions, can directly translate into better post-transplant outcomes.

### 4.4. Study Limitations

This study is based on the Heidelberg HTX Registry and therefore reflects a large, single-center cohort. As with any single-center design, limitations apply, and the conclusions should be interpreted cautiously in view of the broader literature. Nevertheless, the analysis incorporates detailed clinical data from 418 HTX recipients treated and followed according to standardized protocols, thereby mitigating the risk of selection bias and residual confounding [21,22,23].

To ensure adequate statistical power, we included recipients transplanted between 2000 and 2019 with follow-up of up to 5 years. Changes in surgical and medical practices over time may have influenced outcomes after HTX (era effects). To account for this, we conducted a sensitivity analysis limited to HTX recipients receiving mycophenolic acid and tacrolimus, the maintenance immunosuppressive regimen at our center since 2006. The concordance of these findings with the main analysis supports the overall robustness of our study results [21,22,23].

Despite these limitations, this study is, to our knowledge, one of the largest to date to demonstrate that an elevated pre-transplant CRP is an independent predictor of mortality after HTX. Although causality cannot be inferred, the strength and specificity of the association to infection-related deaths support a biologically plausible connection that merits further confirmation in prospective, multicenter studies.

## 5. Conclusions

In this retrospective, single-center cohort of 418 HTX recipients, elevated pre-transplant CRP (≥20 mg/L) was associated with significantly increased short- and long-term mortality after HTX. Excess 5-year mortality after HTX was largely driven by infection- and sepsis-related deaths, particularly pulmonary infections. Pre-transplant CRP ≥ 20 mg/L remained an independent predictor of 5-year mortality after HTX in multivariate analysis and was consistently associated with reduced post-transplant survival across calcineurin inhibitor regimens, with no post-transplant survival difference between cyclosporine A and tacrolimus in this high-risk group. Pre-transplant CRP ≥ 20 mg/L may therefore serve as a practical cut-off to identify a high-risk subgroup of HTX candidates who may benefit from intensified pre- and post-transplant infectious risk assessment and surveillance.

## Figures and Tables

**Table 1:** Demographic and clinical characteristics.

Parameter	All Patients (*n* = 418)	CRP < 20 mg/L Before HTX (*n* = 316)	CRP ≥ 20 mg/L Before HTX (*n* = 102)	Difference	95% CI	*p*-Value
Recipient data						
Age (years), mean ± SD	52.1 ± 10.2	52.0 ± 10.0	52.4 ± 10.7	0.4	−1.9–2.7	*0.696*
Male sex, *n* (%)	317 (75.8%)	236 (74.7%)	81 (79.4%)	4.7%	−4.5–13.9%	*0.332*
BMI (kg/m^2^), mean ± SD	25.3 ± 4.3	25.2 ± 4.4	25.5 ± 4.1	0.3	−0.6–1.2	*0.489*
Arterial hypertension, *n* (%)	231 (55.3%)	174 (55.1%)	57 (55.9%)	0.8%	−10.3–11.9%	*0.885*
Dyslipidemia, *n* (%)	267 (63.9%)	203 (64.2%)	64 (62.7%)	1.5%	−9.3–12.3%	*0.785*
Diabetes mellitus, *n* (%)	131 (31.3%)	94 (29.7%)	37 (36.3%)	6.6%	−4.0–17.2%	*0.217*
Peripheral artery disease, *n* (%)	35 (8.4%)	26 (8.2%)	9 (8.8%)	0.6%	−5.7–6.9%	*0.850*
COPD, *n* (%)	105 (25.1%)	73 (23.1%)	32 (31.4%)	8.3%	−1.8–18.4%	*0.094*
Chronic kidney disease ^, *n* (%)	230 (55.0%)	161 (50.9%)	69 (67.6%)	16.7%	6.1–27.3%	0.003 *
eGFR (ml/min/1.73 m^2^), mean ± SD	59.6 ± 23.5	61.8 ± 23.2	52.7 ± 23.3	9.1	3.9–14.3	0.001 *
Open-heart surgery before HTX						
Overall open-heart surgery, *n* (%)	132 (31.6%)	95 (30.1%)	37 (36.3%)	6.2%	−4.4–16.8%	*0.241*
CABG surgery, *n* (%)	49 (11.7%)	39 (12.3%)	10 (9.8%)	2.5%	−4.3–9.3%	*0.488*
Other surgery °, *n* (%)	43 (10.3%)	29 (9.2%)	14 (13.7%)	4.5%	−2.9–11.9%	*0.189*
VAD surgery, *n* (%)	52 (12.4%)	35 (11.1%)	17 (16.7%)	5.6%	−2.4–13.6%	*0.137*
Primary indication for HTX						
Ischemic CMP, *n* (%)	136 (32.5%)	108 (34.2%)	28 (27.4%)	6.8%	−3.3–16.9%	*0.207*
Non-ischemic CMP, *n* (%)	209 (50.0%)	151 (47.8%)	58 (56.9%)	9.1%	−2.0–20.2%	*0.111*
Valvular heart disease, *n* (%)	16 (3.8%)	9 (2.8%)	7 (6.9%)	4.1%	−1.1–9.3%	*0.066*
Cardiac amyloidosis, *n* (%)	57 (13.7%)	48 (15.2%)	9 (8.8%)	6.4%	−0.4–13.2%	*0.103*
Donor data						
Age (years), mean ± SD	44.3 ± 12.7	44.4 ± 12.4	43.8 ± 13.6	0.6	−2.4–3.6	*0.694*
Male sex, *n* (%)	143 (34.2%)	103 (32.6%)	40 (39.2%)	6.6%	−4.2–17.4%	*0.220*
BMI (kg/m^2^), mean ± SD	25.1 ± 4.5	25.1 ± 4.7	25.1 ± 4.1	0.0	−1.0–1.0	*0.999*
Donor–recipient sex mismatch						
Mismatch, *n* (%)	205 (49.0%)	158 (50.0%)	47 (46.0%)	4.0%	−7.1–15.1%	*0.491*
Donor (m) to recipient (f), *n* (%)	15 (3.6%)	12 (3.8%)	3 (2.9%)	0.9%	−3.0–4.8%	*0.686*
Donor (f) to recipient (m), *n* (%)	190 (45.4%)	146 (46.2%)	44 (43.1%)	3.1%	−8.0–14.2%	*0.589*
Perioperative data						
Ischemic time (min), mean ± SD	251.3 ± 59.1	247.6 ± 57.9	262.6 ± 61.7	15.0	1.4–28.6	0.032 *
Biatrial anastomosis, *n* (%)	5 (1.2%)	4 (1.3%)	1 (1.0%)	0.3%	−2.0–2.6%	*0.818*
Bicaval anastomosis, *n* (%)	184 (44.0%)	141 (44.6%)	43 (42.1%)	2.5%	−8.5–13.5%	*0.663*
Total orthotopic anastomosis, *n* (%)	229 (54.8%)	171 (54.1%)	58 (56.9%)	2.8%	−8.3–13.9%	*0.628*

BMI = body mass index; CABG = coronary artery bypass graft; CI = confidence interval; CMP = cardiomyopathy; COPD = chronic obstructive pulmonary disease; CRP = C-reactive protein; f = female; eGFR = estimated glomerular filtration rate; HTX = heart transplantation; m = male; *n* = number; SD = standard deviation; VAD = ventricular assist device; ^ = eGFR < 60 mL/min/1.73 m^2^; ° = congenital, valvular, or ventricular surgery; * = statistically significant (*p* < 0.050).

**Table 2:** Post-transplant medications.

Parameter	All Patients (*n* = 418)	CRP < 20 mg/L Before HTX (*n* = 316)	CRP ≥ 20 mg/L Before HTX (*n* = 102)	Difference	95% CI	*p*-Value
Immunosuppressive drug therapy						
Cyclosporine A, *n* (%)	126 (30.1%)	100 (31.6%)	26 (25.5%)	6.1%	−3.8–16.0%	0.239
Tacrolimus, *n* (%)	292 (69.9%)	216 (68.4%)	76 (74.5%)	6.1%	−3.8–16.0%	0.239
Azathioprine, *n* (%)	46 (11.0%)	37 (11.7%)	9 (8.8%)	2.9%	−3.6–9.4%	0.418
Mycophenolic acid, *n* (%)	372 (89.0%)	279 (88.3%)	93 (91.2%)	2.9%	−3.6–9.4%	0.418
Steroids, *n* (%)	418 (100.0%)	316 (100.0%)	102 (100.0%)	0.0%	n. a.	n. a.
Concomitant medications						
ASA, *n* (%)	59 (14.1%)	47 (14.9%)	12 (11.8%)	3.1%	−4.3–10.5%	0.433
Beta blocker, *n* (%)	93 (22.2%)	75 (23.7%)	18 (17.6%)	6.1%	−2.7–14.9%	0.199
Ivabradine, *n* (%)	61 (14.6%)	43 (13.6%)	18 (17.6%)	4.0%	−4.3–12.3%	0.315
Calcium channel blocker, *n* (%)	128 (30.6%)	100 (31.6%)	28 (27.5%)	4.1%	−6.0–14.2%	0.424
ACE inhibitor/ARB, *n* (%)	175 (41.9%)	139 (44.0%)	36 (35.3%)	8.7%	−2.1–19.5%	0.122
Diuretic, *n* (%)	418 (100.0%)	316 (100.0%)	102 (100.0%)	0.0%	n. a.	n. a.
Statin, *n* (%)	240 (57.4%)	188 (59.5%)	52 (51.0%)	8.5%	−2.6–19.6%	0.131
Oral anti-diabetic therapy, *n* (%)	72 (17.2%)	51 (16.1%)	21 (20.6%)	4.5%	−4.3–13.3%	0.301
Insulin therapy, *n* (%)	64 (15.3%)	45 (14.2%)	19 (18.6%)	4.4%	−4.1–12.9%	0.285
Gastric protection drug ^†^, *n* (%)	418 (100.0%)	316 (100.0%)	102 (100.0%)	0.0%	n. a.	n. a.

ACE inhibitor = angiotensin-converting enzyme inhibitor; ARB = angiotensin II receptor blocker; ASA = acetylsalicylic acid; CI = confidence interval; CRP = C-reactive protein; *n* = number; n. a. = not applicable; ^†^ = gastric protection drug defined as proton pump inhibitor (PPI) or histamine receptor (H_2_) blocker.

**Table 3:** Post-transplant primary outcome.

Parameter	All Patients (*n* = 418)	CRP < 20 mg/L Before HTX (*n* = 316)	CRP ≥ 20 mg/L Before HTX (*n* = 102)	Difference	95% CI	*p*-Value
30-day mortality after HTX, *n* (%)	28 (6.7%)	16 (5.1%)	12 (11.8%)	6.7%	0.1–13.3%	0.019 *
1-year mortality after HTX, *n* (%)	95 (22.7%)	55 (17.4%)	40 (39.2%)	21.8%	11.4–32.2%	<0.001 *
2-year mortality after HTX, *n* (%)	116 (27.8%)	73 (23.1%)	43 (42.2%)	19.1%	8.4–29.8%	<0.001 *
5-year mortality after HTX, *n* (%)	144 (34.4%)	96 (30.4%)	48 (47.1%)	16.7%	5.8–27.6%	0.002 *

CI = confidence interval; CRP = C-reactive protein; HTX = heart transplantation; *n* = number; * = statistically significant (*p* < 0.050).

**Table 4:** Causes of death within 5 years after HTX.

Parameter	All Patients (*n* = 418)	CRP < 20 mg/L Before HTX (*n* = 316)	CRP ≥ 20 mg/L Before HTX (*n* = 102)	Difference	95% CI	*p*-Value
Graft failure, *n* (%)	40 (9.6%)	29 (9.2%)	11 (10.8%)	1.6%	−5.2–8.4%	0.631
Acute rejection, *n* (%)	5 (1.2%)	4 (1.3%)	1 (1.0%)	0.3%	−2.0–2.6%	0.818
Infection/Sepsis, *n* (%)	79 (18.9%)	50 (15.8%)	29 (28.4%)	12.6%	3.0–22.2%	0.005 *
Pulmonary infection, *n* (%)	50 (12.0%)	30 (9.5%)	20 (19.6%)	10.1%	1.7–18.5%	0.006 *
Abdominal infection, *n* (%)	29 (6.9%)	20 (6.3%)	9 (8.8%)	2.5%	−3.6–8.6%	0.389
Malignancy, *n* (%)	9 (2.1%)	7 (2.2%)	2 (2.0%)	0.2%	−3.0–3.4%	0.878
Thromboembolic event/bleeding, *n* (%)	11 (2.6%)	6 (1.9%)	5 (4.9%)	3.0%	−1.5–7.5%	0.099
All causes, *n* (%)	144 (34.4%)	96 (30.4%)	48 (47.1%)	16.7%	5.8–27.6%	0.002 *

CI = confidence interval; CRP = C-reactive protein; HTX = heart transplantation; *n* = number; * = statistically significant (*p* < 0.050).

**Table 5:** Multivariate analysis for 5-year mortality after HTX.

Parameter	Hazard Ratio	95% CI	*p*-Value
Chronic kidney disease ^ (in total)	1.337	0.808–2.213	0.258
eGFR (ml/min/1.73 m^2^)	0.993	0.982–1.004	0.195
Ischemic time (min)	1.002	0.999–1.005	0.168
CRP ≥ 20 mg/L before HTX (in total)	1.630	1.144–2.323	0.007 *

CI = confidence interval; CRP = C-reactive protein; eGFR = estimated glomerular filtration rate; HTX = heart transplantation; ^ = eGFR < 60 mL/min/1.73 m^2^; * = statistically significant (*p* < 0.050).

**Table 6:** Post-transplant secondary outcomes.

Parameter	All Patients (*n* = 418)	CRP < 20 mg/L Before HTX (*n* = 316)	CRP ≥ 20 mg/L Before HTX (*n* = 102)	Difference	95% CI	*p*-Value
30-day atrial fibrillation after HTX, *n* (%)	58 (13.9%)	42 (13.3%)	16 (15.7%)	2.4%	−5.6–10.4%	0.543
30-day rejection episode after HTX, *n* (%)	53 (12.7%)	43 (13.6%)	10 (9.8%)	3.8%	−3.1–10.7%	0.315
30-day TIA after HTX, *n* (%)	0 (0.0%)	0 (0.0%)	0 (0.0%)	0.0%	n. a.	n. a.
30-day stroke after HTX, *n* (%)	15 (3.6%)	9 (2.8%)	6 (5.9%)	3.1%	−1.8–8.0%	0.152

CI = confidence interval; CRP = C-reactive protein; HTX = heart transplantation; *n* = number; n. a. = not applicable; TIA = transient ischemic attack.

## Data Availability

The original contributions presented in this study are included in the article, further inquiries can be directed to the corresponding author.

## References

[1] McDonagh T.A., Metra M., Adamo M., Gardner R.S., Baumbach A., Böhm M., Burri H., Butler J., Čelutkienė J., Chioncel O. (2021). 2021 ESC Guidelines for the diagnosis and treatment of acute and chronic heart failure. Eur. Heart J..

[2] Stehlik J., Kobashigawa J., Hunt S.A., Reichenspurner H., Kirklin J.K. (2018). Honoring 50 Years of Clinical Heart Transplantation in Circulation: In-Depth State-of-the-Art Review. Circulation.

[3] Zhu Y., Lingala B., Baiocchi M., Toro Arana V., Williams K.M., Shudo Y., Oyer P.E., Woo Y.J. (2021). The Stanford experience of heart transplantation over five decades. Eur. Heart J..

[4] Hunt S.A. (2006). Taking heart-cardiac transplantation past, present, and future. N. Engl. J. Med..

[5] Awad M.A., Shah A., Griffith B.P. (2022). Current status and outcomes in heart transplantation: A narrative review. Rev. Cardiovasc. Med..

[6] Ortega-Legaspi J.M., Bravo P.E. (2021). Diagnosis and management of cardiac allograft vasculopathy. Heart.

[7] Crespo-Leiro M.G., Alonso-Pulpón L., Vázquez de Prada J.A., Almenar L., Arizón J.M., Brossa V., Delgado J.F., Fernandez-Yañez J., Manito N., Rábago G. (2008). Malignancy after heart transplantation: Incidence, prognosis and risk factors. Am. J. Transplant..

[8] Moayedi Y., Gomez C.A., Fan C.P.S., Miller R.J.H., Bunce P.E., Tremblay-Gravel M., Foroutan F., Manlhiot C., Yee J., Shullo M.A. (2019). Infectious complications after heart transplantation in patients screened with gene expression profiling. J. Heart Lung Transplant..

[9] Pons S., Sonneville R., Bouadma L., Styfalova L., Ruckly S., Neuville M., Radjou A., Lebut J., Dilly M.P., Mourvillier B. (2019). Infectious complications following heart transplantation in the era of high-priority allocation and extracorporeal membrane oxygenation. Ann. Intensive Care.

[10] Pata R., Kristeva J., Kosuru B. (2024). Pneumonia in Transplant Recipients: A Comprehensive Review of Diagnosis and Management. Cureus.

[11] Jordan A.M., Tatum R., Ahmad D., Patel S.V., Maynes E.J., Weber M.P., Moss S., Royer T.L., Tchantchaleishvili V., Massey H.T. (2022). Infective endocarditis following heart transplantation: A systematic review. Transplant. Rev..

[12] Siani A., Perone F., Costantini P., Rodolfi S., Muscogiuri G., Sironi S., Carriero S., Pavon A.G., van der Bilt I., van Rosendael P. (2022). Aortic regurgitation: A multimodality approach. J. Clin. Ultrasound.

[13] Burger P.M., Koudstaal S., Mosterd A., Fiolet A.T.L., Teraa M., van der Meer M.G., Cramer M.J., Visseren F.L.J., Ridker P.M., Dorresteijn J.A.N. (2023). C-Reactive Protein and Risk of Incident Heart Failure in Patients With Cardiovascular Disease. J. Am. Coll. Cardiol..

[14] Varagunam M., Finney H., Trevitt R., Sharples E., McCloskey D.J., Sinnott P.J., Raftery M.J., Yaqoob M.M. (2004). Pretransplantation levels of C-reactive protein predict all-cause and cardiovascular mortality, but not graft outcome, in kidney transplant recipients. Am. J. Kidney Dis..

[15] Kim Y.K., Kim S.H., Lee S.D., Hong S.K., Park S.J. (2015). Pretransplant serum levels of C-reactive protein predict prognoses in patients undergoing liver transplantation for hepatocellular carcinoma. Transplant. Proc..

[16] Wu P., Liang W., Chen X., Chen L., Yang X., Yan Z., Wang W. (2019). Pretransplant C-reactive protein as a prognostic marker in allogeneic stem cell transplantation: A PRISMA-compliant meta-analysis. Medicine.

[17] Huma L., Suciu H., Avram C., Suteu R.A., Danilesco A., Baba D.F., Moldovan D.A., Sin A.I. (2024). Implications of Preoperative C-Reactive Protein Levels in Heart Transplant Patients-A Single-Center Retrospective Study. J. Clin. Med..

[18] Mehra M.R., Kobashigawa J., Starling R., Russell S., Uber P.A., Parameshwar J., Mohacsi P., Augustine S., Aaronson K., Barr M. (2006). Listing criteria for heart transplantation: International Society for Heart and Lung Transplantation guidelines for the care of cardiac transplant candidates—2006. J. Heart Lung Transplant..

[19] Mehra M.R., Canter C.E., Hannan M.M., Semigran M.J., Uber P.A., Baran D.A., Danziger-Isakov L., Kirklin J.K., Kirk R., Kushwaha S.S. (2016). The 2016 International Society for Heart Lung Transplantation listing criteria for heart transplantation: A 10-year update. J. Heart Lung Transplant..

[20] Peled Y., Ducharme A., Kittleson M., Bansal N., Stehlik J., Amdani S., Saeed D., Cheng R., Clarke B., Dobbels F. (2024). International Society for Heart and Lung Transplantation Guidelines for the Evaluation and Care of Cardiac Transplant Candidates-2024. J. Heart Lung Transplant..

[21] Heil K.M., Rivinius R., Helmschrott M., Rahm A.K., Ehlermann P., Frey N., Darche F.F. (2025). Increased Pre-Transplant Carotid Intima-Media Thickness Is Associated with Early Post-Transplant Atrial Fibrillation, Stroke, and Reduced Survival After Heart Transplantation. Life.

[22] Heil K.M., Rivinius R., Helmschrott M., Rahm A.K., Ehlermann P., Frey N., Darche F.F. (2025). Pre-Transplant Heavy Smoking Is Associated with Reduced Survival After Heart Transplantation Due to Infection and Malignancy. J. Clin. Med..

[23] Rivinius R., Helmschrott M., Ruhparwar A., Schmack B., Darche F.F., Thomas D., Bruckner T., Katus H.A., Ehlermann P., Doesch A.O. (2018). COPD in patients after heart transplantation is associated with a prolonged hospital stay, early posttransplant atrial fibrillation, and impaired posttransplant survival. Clin. Epidemiol..

[24] Eccles S., Pincus C., Higgins B., Woodhead M., Guideline Development Group (2014). Diagnosis and management of community and hospital acquired pneumonia in adults: Summary of NICE guidance. BMJ.

[25] Biasucci L.M., Liuzzo G., Grillo R.L., Caligiuri G., Rebuzzi A.G., Buffon A., Summaria F., Ginnetti F., Fadda G., Maseri A. (1999). Elevated levels of C-reactive protein at discharge in patients with unstable angina predict recurrent instability. Circulation.

[26] Haverkate F., Thompson S.G., Pyke S.D., Gallimore J.R., Pepys M.B. (1997). Production of C-reactive protein and risk of coronary events in stable and unstable angina. European Concerted Action on Thrombosis and Disabilities Angina Pectoris Study Group. Lancet.

[27] Liuzzo G., Biasucci L.M., Gallimore J.R., Grillo R.L., Rebuzzi A.G., Pepys M.B., Maseri A. (1994). The prognostic value of C-reactive protein and serum amyloid a protein in severe unstable angina. N. Engl. J. Med..

[28] Kirklin J.K., Naftel D.C., Bourge R.C., McGiffin D.C., Hill J.A., Rodeheffer R.J., Jaski B.E., Hauptman P.J., Weston M., White-Williams C. (2003). Evolving trends in risk profiles and causes of death after heart transplantation: A ten-year multi-institutional study. J. Thorac. Cardiovasc. Surg..

[29] Schaenman J., Goldwater D. (2020). The aging transplant population and immunobiology: Any therapeutic implication?. Curr. Opin. Organ. Transplant..

[30] Giovannico L., Santobuono V.E., Fischetti G., Mazzone F., Parigino D., Savino L., Alfeo M., Milano A.D., Guaricci A.I., Ciccone M.M. (2025). Kinetics of Procalcitonin, CRP, IL-6, and Presepsin in Heart Transplant Patients Undergoing Induction with Thymoglobulin (rATG). J. Clin. Med..

[31] Drozd M., Pujades-Rodriguez M., Morgan A.W., Lillie P.J., Witte K.K., Kearney M.T., Cubbon R.M. (2022). Systemic Inflammation Is Associated With Future Risk of Fatal Infection: An Observational Cohort Study. J. Infect. Dis..

[32] Helmschrott M., Rivinius R., Ruhparwar A., Schmack B., Erbel C., Gleissner C.A., Akhavanpoor M., Frankenstein L., Ehlermann P., Bruckner T. (2015). Advantageous effects of immunosuppression with tacrolimus in comparison with cyclosporine A regarding renal function in patients after heart transplantation. Drug Des. Dev. Ther..

[33] Helmschrott M., Rivinius R., Bruckner T., Katus H.A., Doesch A.O. (2017). Renal function in heart transplant patients after switch to combined mammalian target of rapamycin inhibitor and calcineurin inhibitor therapy. Drug Des. Dev. Ther..

[34] Lund L.H., Edwards L.B., Kucheryavaya A.Y., Dipchand A.I., Benden C., Christie J.D., Dobbels F., Kirk R., Rahmel A.O., Yusen R.D. (2013). The Registry of the International Society for Heart and Lung Transplantation: Thirtieth Official Adult Heart Transplant Report—2013; focus theme: Age. J. Heart Lung Transplant..

[35] Lund L.H., Edwards L.B., Kucheryavaya A.Y., Benden C., Christie J.D., Dipchand A.I., Dobbels F., Goldfarb S.B., Levvey B.J., Meiser B. (2014). The registry of the International Society for Heart and Lung Transplantation: Thirty-first official adult heart transplant report—2014; focus theme: Retransplantation. J. Heart Lung Transplant..

[36] Eisenberg M.S., Chen H.J., Warshofsky M.K., Sciacca R.R., Wasserman H.S., Schwartz A., Rabbani L.E. (2000). Elevated levels of plasma C-reactive protein are associated with decreased graft survival in cardiac transplant recipients. Circulation.

[37] Labarrere C.A., Woods J.R., Hardin J.W., Jaeger B.R., Zembala M., Deng M.C., Kassab G.S. (2014). Early inflammatory markers are independent predictors of cardiac allograft vasculopathy in heart-transplant recipients. PLoS ONE.

[38] Battes L.C., Caliskan K., Rizopoulos D., Constantinescu A.A., Robertus J.L., Akkerhuis M., Manintveld O.C., Boersma E., Kardys I. (2015). Repeated measurements of NT-pro-B-type natriuretic peptide, troponin T or C-reactive protein do not predict future allograft rejection in heart transplant recipients. Transplantation.

[39] Sezgin Y., Bulut Ş., Bozalıoğlu S., Sezgin A. (2019). Levels of High-Sensitivity C-Reactive Protein in Heart Transplant Patients With and Without Periodontitis. Exp. Clin. Transplant..

